# Restaurants’ compliance with calorie labeling policies in food delivery applications

**DOI:** 10.3389/fnut.2023.1281293

**Published:** 2023-12-14

**Authors:** Abdulaziz S. Alangari

**Affiliations:** ^1^Department of Epidemiology and Biostatistics, College of Public Health and Health Informatics, King Saud bin Abdulaziz University for Health Sciences, Riyadh, Saudi Arabia; ^2^King Abdullah International Medical Research Center, Riyadh, Saudi Arabia

**Keywords:** calorie, food delivery, restaurant compliance, nutrition, public health, policy

## Abstract

**Background:**

To encourage consumers to make healthier choices, the Saudi Food and Drug Authority (SFDA) released the Saudi Arabia Nutrition Labeling Policy, which requires restaurants to present caloric information clearly on their menus. Food delivery applications are also mandated to present caloric information on their platforms. The aim of this study is to explore how restaurants on food delivery applications comply with the calorie labeling policy.

**Methods:**

Data were extracted from a widely used food delivery application in Saudi Arabia to include a sample of 120 “healthy food” restaurants. The restaurants were checked for compliance on both the application and the website. Descriptive and logistic regression analyses were performed to examine the distribution and association of relevant factors.

**Results:**

A substantial proportion (43.3%) of healthy foods on delivery applications (*n* = 120) do not comply with the SFDA calorie labeling policies. Among these restaurants, 56.5% presented the calorie labeling on their menu item. Of those who provided calorie information on their websites (*n* = 62), 54.8% provided appropriate calorie labeling based on the SFDA policy. There was an association between compliance and the number of restaurant categories but no associations for website calorie labeling, rating, and appropriateness.

**Conclusion:**

This study provides important findings for policymakers that will enable them to reinforce food calorie policies on food delivery platforms, restaurant websites, social media, and marketing campaigns. Not all restaurants comply with the SFDA calorie labeling policies. Suggestions to present the calorie-related words in Arabic and offer more information to help the consumer make the appropriate food selection decision. Further studies are needed to explore the reasons for and barriers to posting nutritional information on menu items.

## 1 Introduction

In recent decades, the food industry has grown and restaurant expenditures have increased consistently. In 2022, food spending totaled USD 2.39 trillion in the United States alone (1). Of this, USD 1.16 trillion was spent on away-from-home food, with a 16% increase from 2021. Moreover, worldwide, the market for food delivery stands at EUR 83 billion, which accounts for 4% of the food sold through restaurants and fast-food chains (2). There is a high demand for food delivery services that mobile apps to increase restaurant sales revenue (3). The overall annual growth has increased during the COVID-19 pandemic due to the health precautions taken by the public (4), and it is expected to continue increasing in the years to come (5). This has raised concerns about dietary quality because takeaway and restaurant foods tend to have higher energy, total and saturated fat, and sodium content, and less fiber than foods prepared inside the home (6–8).

The menu labeling policy is part of a wider governmental strategy to tackle obesity and promote healthy eating. In the United States, a similar policy requires food establishments with more than 20 locations to present calorie counts that are easily visible to customers (9). The primary purposes of the policy are to increase health awareness around the recommended energy intake and improve dietary quality, in order to help customers make healthier food choices. Further, it encourages restaurants to reformulate menu items to improve their nutrient profiles (10). The effect of calorie labeling on consumer food choices is mixed. Studies have shown that the implementation of this policy has led some restaurant chains to reduce the calorie content of their menu items (10, 11). Moreover, it was found that calorie labeling alone can nudge individuals to make lower-calorie food and beverage choices from a menu (12, 13). On the other hand, some studies show that calorie labeling is not effective in promoting healthy food choices (14).

The Saudi Food and Drug Authority (SFDA) released the Saudi Arabia Nutrition Labeling Policy as part of the Healthy Food Regulation Strategy to voluntary pledges in 2017, and it was enforced in 2019 (15). The SFDA organized workshops and launched successive awareness campaigns and a guidebook directed to restaurant and café owners to introduce how to calculate and set calories in restaurants’ food menus. The policy requires all restaurants (regardless of size and number of outlets), including dine-in restaurants, fast-food restaurants, and others, to post caloric information clearly on their menus (16). The calorie information must be presented in an easily readable format and must provide calorie information for all items on the menu. In addition, a statement such as “adults need an average of 2,000 calories per day, and individual calorie needs vary from one person to another” should be displayed. In 2023, the policy was updated to include food delivery apps (17). Studies in Saudi Arabia have found that consumers have some understanding of calorie labeling, and they expressed a positive attitude toward it (18, 19). Barriers to providing calorie information might be related to difficulties in calculations, additional costs and planning, information overload, libel risks, and effects on revenue (20).

In Saudi Arabia, almost 60% of the population is either overweight or obese. The prevalence of overweight is higher in males (43%) than in females (33%) while the prevalence of obesity is higher in females (21%) than in males (19%) (21). Aside from physical inactivity and sedentary behaviors, the consumption of unhealthy and calorie-dense food has significantly contributed to that increase (22). The market for eating out is in high demand. According to the Saudi Central Bank reports, restaurants and cafes are contributing to nearly 15% of all point-of-sale transactions (23). The majority of food-delivery-app users (63%) are aged 19–29 years old (24). In addition, it has been reported that 48.4% of users of online food delivery services are aged between 18 and 34 years (4). Moreover, most Saudi citizens are on the younger side, with the average age of a Saudi citizen being 29 years old (25). This indicates that the majority of the Saudi population has the potential to utilize food delivery apps.

To comply with the government’s policy, food delivery apps are required to present nutritional facts, including calories, on their platforms. To this end, some food delivery apps have taken measures to display caloric information on their platforms, such as adding a section on their application that provides calorie-related information. Nevertheless, it has been noted that not all restaurants on food delivery applications have been able to meet the requirements of the policy. This study aims to explore how restaurants on food delivery applications comply with the calorie labeling policy.

## 2 Materials and methods

### 2.1 Sample

There are 36 licensed food delivery applications in Saudi Arabia (26). However, not all of these companies are currently functioning, provide an option for restaurants to insert calorie-related information, or are popular among users. The data were extracted from one of the most widely used food delivery applications in Saudi Arabia through the following procedure. (A) Since Riyadh city is a large city with an area of around 3,100 square kilometers, the address to which the food would be delivered was located in the middle of the city, to ensure that it covers the most geographically distributed restaurants or branches. (B) Data extraction was conducted at different times of the day through April 2023 to ensure the availability of the restaurant opening times. Some restaurants close at certain times, which may result in these restaurants being excluded. (C) As restaurants started to add healthy menu items to meet the increased demand from consumers (27), and to be more conservative to not overestimate the compliance percentages, a filter was applied to include only restaurants classified as serving “healthy food.” One hundred and 41 restaurants (*n* = 141) were found in the application after applying the criteria, and, after careful review, 21 restaurants were excluded because they either do not have any entry or are not a restaurant (such as grocery stores, etc.). The final sample included in the study comprises 120 restaurants.

### 2.2 Measures

Application compliance: for each restaurant, the menu items were checked for compliance by evaluating whether the calorie information was presented. Restaurants that did not include calorie information for all menu items were classified as non-compliant. Restaurants that did not label at least one menu item were classified as partially compliant. Lastly, restaurants that labeled all menu items were classified as compliant. The compliance evaluations were carried out based on the following procedures. (A) Any menu item with no price or “SAR 0” was excluded from the evaluation since it was considered unavailable. (B) Soft drinks were excluded from the evaluation as they can be found anywhere, and it is not mandatory to present their calorie information. However, drinks that were custom-made in the restaurants counted toward the evaluation. (C) Menu items comprising “box or bundle items,” be it a pre-set bundle or a custom-made one, were excluded from the evaluation. (D) Temporary menu items, such as “limited menu items” or “limited offer bundles,” were not included in the evaluation.

Website compliance: restaurants were also assessed with the same classification and evaluation criteria to determine whether their menu items were labeled on their websites. If the restaurant had no official website, then the social media accounts were considered their official public sites. If none (e.g., cloud kitchen), then the restaurant is removed from the respective analyses.

Appropriate labeling: among the restaurants with partially compliant and compliant website menus, further evaluations were conducted to check whether the calorie labeling was presented according to the SFDA policies. These criteria include the font size, appropriate colors, language considerations, and the location on the website (e.g., hidden or in a different menu).

Restaurant rating: the restaurants’ customer ratings (out of 5) were also included in the analysis to determine whether there is an association between the ratings and compliance. These ratings were provided by the customers who use the application. Ratings of “0″ were removed from the analysis because after validating the restaurants it appeared as a newly opened restaurant.

Total restaurant categories: The food delivery application provides multiple food categories in the filter option tab. These are Salads, Bakery, Desserts, Healthy food, Fast food, Arabic, Italian, American, Mexican, Indian, Japanese, Korean, Asian, Lebanese, International, Shawarma and doner, Breakfast, Pizza, Pasta, Seafood, Sushi, Noodles, Sandwiches, Pastries, Juices, Beverages, Grill, Burgers, Vegetarian, and Coffee. The filter was applied to “Healthy food.” Some restaurants may have more than one category and were classified as 1–2 and 3 or more categories.

### 2.3 Statistical analysis

Sample descriptive statistics were computed according to frequency and percentages for categorical data and the mean with its standard deviation for continuous data. To explore variable associations with application compliance, logistic regression models were used to compute odds ratios (OR) and 95% confidence intervals (CI). Significance was set at a *p*-value of less than 0.05. The analyses were conducted using SAS software 9.4, SAS Institute Inc., Cary, NC, United States.

## 3 Results

Among all of the restaurants included in the study (*n* = 120), 43.3% do not comply with the food calorie policies, 32.5% are partially compliant, and only 24.2% are fully compliant (Table 1). The average consumer rating for all restaurants labeled as “healthy food” was 4.2 out of 5, with a standard deviation of 0.4. The ratings were similar across all types of compliance and no differences were observed (compliant, mean = 4.2, ±SD = 0.3; partially compliant, mean = 4.2, ±SD = 0.4; and non-compliant, mean = 4.2, ±SD = 0.4). Moreover, the majority of restaurants (51.7%) tag their food items with one or two types of food categories (including the healthy food category).

**Table 1:** Delivery app menu calorie labeling according to compliance category.

	Non-compliant		Partially compliant		Compliant		Total	
Variable	Mean	±SD	Mean	±SD	Mean	±SD	Mean	±SD
Rating	4.2	0.4	4.2	0.4	4.2	0.3	4.2	0.4
Variable	***n***	**%**	***n***	**%**	***n***	**%**	***n***	**%**
Total categories	52	43.3	39	32.5	29	24.2	120	100.0
1–2	22	42.3	23	59.0	17	58.6	62	51.7
3–4	18	34.6	12	30.8	11	37.9	41	34.2
5 or more	12	23.1	4	10.3	1	3.5	17	14.2
Website calorie labeling	39	42.4	32	34.8	21	22.8	92	76.7
Non-compliant	12	30.8	13	40.6	5	23.8	30	32.6
Partial compliant	7	18.0	2	6.3	1	4.8	10	10.9
Compliant	20	51.3	17	53.1	15	71.4	52	56.5
Website appropriate	27	43.6	19	30.7	16	25.8	62	51.7
Yes	15	44.1	10	29.4	9	26.5	34	54.8
No	12	42.9	9	32.1	7	25.0	28	45.2
Total	52	43.3	39	32.5	29	24.2	120	100.0

Ninety-two (76.7%) of the restaurants have a formal website or a social media account where they present their food menu (Table 1). Of these, the majority (56.5%) are compliant, 32.6% are non-compliant, and 10.9% are partially compliant. Among the restaurants that have a website, 42.4% are non-compliant in the food delivery application, 34.8% are partially compliant, and 22.8% are compliant. The majority of restaurants (54.8%) that were deemed to be compliant or partially compliant on their websites were classified as presenting their calorie information correctly.

Regarding the relationship between the observed factors, some patterns were identified in the regression models; however, the results for the unadjusted models were not statistically significant. Restaurants that are non-compliant on their websites are less likely to be non-compliant in the food delivery application than the compliant restaurants (OR = 0.86; CI = 0.36–2.10; value of *p* = 0.747; Table 2). After adjusting for consumer rating, the suitability of the website labels, and total categories, more precise results emerged. For every one-point increase in the consumer rating, it is almost 70% less likely for the restaurant to be non-compliant (AOR = 0.32; CI = 0.09–1.16; value of *p* = 0.082). Those restaurants with inappropriately presented calorie information on their websites were less likely to be non-compliant in the application than those who stated the calorie information appropriately (AOR = 0.72; CI = 0.24–2.17; value of *p* = 0.557). Restaurants with three or more food categories were more likely to be non-compliant in the food delivery application than those with only one or two categories (AOR = 3.14; CI = 1.02–9.68; value of *p* = 0.047).

**Table 2:** Relationship between factors and calorie compliance on the food delivery application.

	Non-compliant vs. any level of compliance					
	Unadjusted			Adjusted		
Variable	OR	(95% CI)	Value of *p*	AOR	(95% CI)	Value of *p*
Rating	0.64	(0.25–1.65)	0.357	0.32	(0.09–1.16)	0.082
Website calorie labeling			0.747			
Non-compliant	0.86	(0.36–2.10)			NA	
Any level of compliance	1					
Website appropriateness			0.921			0.557
Appropriate	1			1		
Not appropriate	0.95	(0.35–2.61)		0.72	(0.24–2.17)	
Total categories			0.074			0.047^*^
1–2	1			1		
3 and more	1.95	(0.94–4.05)		3.14	(1.02–9.68)	

OR, odds ratio; AOR, adjusted odds ratio; CI, confidence intervals; ^*^Significant at value of *p* < 0.05; NA, not applicable.

## 4 Discussion

Obesity is an ongoing public health threat that not only has consequences for the individual but also places a burden on the economy and health systems (28). This is why governments have started to take preventive measures, such as food calorie labeling policies, to support consumer health by making it easier for them to choose more healthy items. The results of this study show that nearly a quarter (24.2%) of restaurants that provided healthy food options fully comply with the food calorie labeling policy in food delivery applications. A similar study in Canada found that 15.4%–53.8% of restaurants presented calorie information for their food (29). However, in some regions, presenting the food calorie information was not mandatory.

The results show no significant difference between the average consumer restaurant ratings and the compliance category. Consumer ratings are a major component of E-commerce business. Although such ratings can be complex, inaccurate, unstandardized, and biased, they give both consumers and business owners a form of feedback and allow different perspectives to be expressed (30). The food quality, service, price, atmosphere, and location are the main factors that influence restaurant ratings (31). Ratings given in the app do not necessarily reflect the restaurant’s overall quality, as the ratings might depend on a specific food item. If a consumer is satisfied with the meal they ordered, they will rate the restaurant itself not that specific meal or menu item. This creates a faulty generalization as other menu items might be not necessarily satisfactory. The application does not have a way to rate a specific menu item or have a comments tab. Also, the major difference between ordering food from a restaurant and ordering it through a third party (a food delivery app, in this case) is the direct contact with the restaurant. This may influence the ratings in the sense that the overall evaluation of the restaurant can, in part, be validated through the experience of visiting the restaurant, through factors such as employee attitudes, restaurant cleanliness, and presentation (32).

The majority of restaurants (51.7%) label their food items with one or two types of food categories only (including the healthy food category). Notably, restaurants with three or more food categories are three times more likely to be non-compliant in the food delivery application than those with one or two categories. It cannot be determined what sort of association is represented in this regard. However, the more food categories, the more varied the food items are in the restaurant. The problem here is that restaurants might not necessarily aim to be healthy restaurants, but they add healthy options to meet the consumer demand for healthy foods (27). This leads the restaurant to be labeled “healthy food” in the food delivery app. Despite this, it might be difficult for the restaurant to calculate the calorie information if they have a wide range of food items on their menu, especially if it is done without the consultation of a specialist. However, the food labeling calculator tool is an initiative of the SFDA to help food manufacturers calculate their products’ nutritional labels (33). Labeling a restaurant with “healthy food” is sometimes misleading, as there is no clear definition of what “healthy foods” are; this category is therefore unlike other categories, such as ethnic or international foods, which are easily identifiable. This issue has prompted the United States Food and Drug Administration to propose a new definition for so-called healthy foods (34).

There was no association between the labeling of calorie information on the restaurant’s website and the food delivery application. Although having a website is important (35), not all restaurants in this study have a web presence; this might be due to these restaurants having a small business structure or a “cloud kitchen,” where there is a commercial cooking space for takeaways only, rather than a full-service restaurant (36). However, surprisingly, approximately 40% of the restaurants that provide the full calorie information for all menu items on their website do not present this information in the application, or else they present partial information for menu items. This raises a concern about why this information is not provided in the application. Food delivery applications provide a standardized way of presenting calorie information, whereby restaurants can update the restaurant information, such as new food items, calorie information, and current offers, through a specific portal. This information is directly reflected in the application.

This study provides important results for policymakers aiming to enforce the food calorie policy on food delivery platforms. Suggestions include uniformly presenting the word for “calorie” in Arabic and offering more information to help the consumer make appropriate food selection decisions (e.g., a calorie range (min–max), nutritional facts, and other information). Having additional information on restaurant menus, such as sodium content, has been proven to influence consumers’ decisions to favor menu items with lower sodium content (37). Moreover, strategies need to be put in place to reinforce food calorie policies for restaurant websites, social media platforms, food delivery applications, and marketing campaigns. To our knowledge, this is the first study to examine restaurant compliance in relation to presenting calorie information on websites and in food delivery applications. While this study takes a unique approach to evaluating food calorie compliance, it has several limitations. First, as this is a cross-sectional study that explores publicly available data, the limited number of variables obtained from the food delivery application hindered further analyses. Second, the extracted data might not be valid for the factors that were intended to be measured. For example, the rating score and restaurant categories might bias the results for the reasons mentioned previously, and they might not represent reality. In addition, an order can be a combination of multiple menu items including healthy and unhealthy items such as sugary drinks, which might distort the relationship between ratings and restaurant compliance. Third, the number of restaurants in the food delivery application is enormous, and only the subsample of “healthy foods” was examined. The results might differ if other types of restaurants, such as restaurant chains, are included. Unfortunately, there is no clear definition of a restaurant chain.

## 5 Conclusion

A substantial proportion of restaurants that provide healthy food options on delivery applications do not comply with the calorie labeling policy. There is inconsistency between the calorie information posted on restaurants’ websites and in food delivery apps. There is a need to reinforce restaurants’ food calorie policies on food delivery platforms, restaurant websites, and social media, and in marketing campaigns. Food delivery applications should not accept any restaurants during registration until they provide the necessary caloric information. Future research should explore the reasons for and barriers to restaurants posting nutritional information on menu items.

## Data availability statement

The raw data supporting the conclusions of this article will be made available by the author, without undue reservation.

## Ethics statement

The study was approved by King Abdullah International Medical Research Center and ethical review and approval was waived since it does not include human subjects or relevant data (Approval #: NRC23R/448/07).

## Author contributions

AA: Conceptualization, Data curation, Formal analysis, Investigation, Methodology, Visualization, Writing – original draft, Writing – review & editing.

## Appendix

Food category frequency in sample restaurants.

**Table:**

Food category	Frequency
Healthy food	120
Salads	39
Sandwiches	36
Vegetarian	14
Arabic	12
International	11
Breakfast	10
Fast Food	9
Beverages	9
Desserts	8
Asian	7
Juices	6
Burgers	6
American	5
Lebanese	5
Sea Food	5
Grill	5
Italian	4
Pizza	4
Bakery	3
Mexican	3
Noodles	3
Japanese	2
Korean	2
Shawarma Doner	2
Pasta	2
Sushi	2
Indian	1
Coffee	1
Pastries	0
